# EXPLORING THE MEDIATION EFFECT OF SOCIAL SUPPORT ON CAREGIVERS’ PHYSICAL HEALTH FOR PERSONS WITH DEMENTIA

**DOI:** 10.1093/geroni/igae098.3388

**Published:** 2024-12-31

**Authors:** Hannah Cho, Liming Huang, Karla Washington, Debra Parker Oliver, George Demiris

**Affiliations:** University of Pennsylvania, Philadelphia, Pennsylvania, United States; University of Pennsylvania, Philadelphia, Pennsylvania, United States; Washington University in St. Louis, St. Louis, Missouri, United States; Washington University in St. Louis, St. Louis, Missouri, United States; University of Pennsylvania, Philadelphia, Pennsylvania, United States

## Abstract

In the U.S., over 11 million families provide 18 trillion hours of unpaid care yearly to Persons with Dementia (PwD). Families, often primary caregivers, face overwhelming stress and health decline due to caregiving demands and employment. This study examines how social support affects caregivers’ stress and physical health in hospice settings for caregivers of PwD. From September 2022 to February 2024 the parent study (n= 167) was assessed using the Medical Outcomes Survey and the Short-Form 12 Questionnaire. The correlation analysis revealed that social support is positively correlated with caregiving duration and negatively correlated with physical health in hospice care. Consequently, a path analysis was conducted to examine the impact of social support on the relationship between caregiving duration and physical health of caregivers of PwD during hospice care. We controlled for age and gender in regression. Preliminary results showed the mediation effect of social support is 0.12 (p =.12). The direct and total effects of caregiving duration on physical health are 0.04 (p = 0.9) and 0.16 (p =.58), respectively. The model is just identified with an RMSEA of 0 and a TLI and CFI of 1. Non-significant results imply that the mediation effect may not be a substantial component of the total effect or that with the sample size statistical power is not high enough to detect it. Given these findings, further investigation is needed, and the data should be re-run as the intervention is ongoing with the full dataset for more definitive insights.

